# Hypothyroidism and dermato/polymyositis: a two-sample Mendelian randomization study

**DOI:** 10.3389/fendo.2024.1361581

**Published:** 2024-09-04

**Authors:** Qianqian Li, Shaoying Yang, Yan Ma, Huijing Huang, Langxian Zhi, Suli Wang, Liangjing Lu

**Affiliations:** ^1^ Department of Rheumatology, Ren Ji Hospital, Shanghai Jiao Tong University School of Medicine, Shanghai, China; ^2^ Department of Rheumatology, Zhongshan Hospital Fudan University, Shanghai, China

**Keywords:** dermatomyositis, polymyositis, hypothyroidism, Mendelian randomization, immune disease

## Abstract

**Objective:**

Observational studies have revealed a higher probability of hypothyroidism in patients with dermatomyositis (DM) or polymyositis (PM), but there is no consensus on whether hypothyroidism causally influences DM or PM. In the present study, we assessed the causal association between hypothyroidism and the risk of dermatomyositis or polymyositis using two-sample Mendelian randomization (TSMR).

**Methods:**

The genome-wide association data of hypothyroidism and dermatomyositis/polymyositis were obtained from the IEU Open GWAS project. Then, TSMR was used to determine whether hypothyroidism is causally associated with DM or PM. Single-nucleotide polymorphisms (SNPs) significantly associated with hypothyroidism were identified and used as instrumental variables (IVs), and the causal relationship between hypothyroidism and DM/PM was examined using TSMR. MR pleiotropy and Cochran’s Q test were used to confirm the heterogeneity and pleiotropy of identified IVs, then four different models, including the inverse variance weighted model (IVW), MR-Egger, weighted median and weighted model were applied in this MR analysis.

**Results:**

Sixty-eight SNPs for DM and 68 SNPs for PM were selected as the IVs (P<5×10^−8^; linkage disequilibrium R^2^ <0.001) to assess the causal association between hypothyroidism and DM/PM selected from GWASs on hypothyroidism. The results revealed a positive causal effect of hypothyroidism on both DM and PM (DM: OR 2.563, 95% CI [1.348, 4.874], P = 0.00156; PM: OR1.709, 95% CI [1.157, 2.525], P =0.007). Moreover, there was no heterogeneity or pleiotropy in the results.

**Conclusion:**

In conclusion, the MR analysis results provided strong evidence to indicate that hypothyroidism might be causally associated with DM and PM. These findings may have important implications for the pathogenesis and possible future therapies of DM/PM.

## 1 Introduction

Idiopathic inflammatory myositis (IIM) is a type of heterogeneous immune-mediated disease characterized by the chronic inflammation of skeletal muscle. The main clinical manifestations are progressive muscle weakness and muscle pain in the proximal extremities (1). The most common clinical subtypes of IIM in adults are polymyositis (PM) and dermatomyositis (DM) (2, 3), which are characterized by the subacute onset of symmetric proximal muscle weakness, common involvement of other organ systems, such as the lungs and skin, a strong association with autoantibodies, and responsiveness to immunosuppression. Both are widely accepted as having an autoimmune basis. Both genetic and environmental factors can affect the occurrence and development of DM or PM (4), but existing research has not been able to clarify the pathogenesis of DM and PM. Even worse, treatment options in DM/PM are limited thus far. Understanding the comorbidities in these patient populations, particularly their relationship, is essential and can improve clinical practice, including survival and health-related quality of life.

Muscular functionality may be influenced by inflammation but also by the endocrine system. In particular, thyroid hormones play a pivotal role in controlling fiber myogenesis, damage repair and transcription profiles; in individuals with hypothyroidism, muscle functionality markedly decreases (5). Hypothyroidism is an endocrine disease caused by the reduction of thyroid hormone synthesis and secretion or the weakening of tissue function (6). Studies have shown that hypothyroidism is associated with autoimmune diseases (7). In a study conducted in Poland, seven of 28 (25%) DM or PM patients had hypothyroidism as a comorbidity (8), and a large case-control study from Israel of 12,278 patients found that the rate of hypothyroidism was significantly (11.2%) higher and significantly (P < 0.0001) associated with DM/PM (9).

Prior studies have found that hypothyroidism is related to DM/PM, but whether there is a causal connection between them is yet unknown. Evidence of the link between hypothyroidism and DM/PM is scarce and mainly anecdotal, based on case reports or case studies; in addition, there is a lack of relevant data from randomized controlled trials, which are difficult to conduct due to practicality, cost, and ethical considerations. However, inadequate adjustment for potential confounding factors can bias the relationship between hypothyroidism and DM/PM. Therefore, a better approach is needed to assess their causal relationship.

Mendelian randomization (MR) is a technique that uses genetic variants as instrumental variables (IVs) to assess whether an observational association between a risk factor and an outcome is consistent with a causal effect (10). The two-sample MR is a method used to estimate the causal effect of an exposure on an outcome using only summary statistics from genome-wide association studies (GWAS). The genetic variant–risk factor association and the genetic variant–outcome association come from independent study populations. Additionally, it is imperative that the two samples represent similar underlying populations (11). Given the increased statistical power of the two-sample MR method, which can leverage existing aggregated data from large-scale GWAS consortia (12).

In this study, we conducted a two-sample MR analysis using GWAS summary statistics from a publicly available GWAS database to investigate the causal effects of hypothyroidism on the risk of developing DM or PM and to further elucidate the genetic correlation between hypothyroidism and DM/PM.

## 2 Methods

### 2.1 Study design and participants

**Figure 1:**
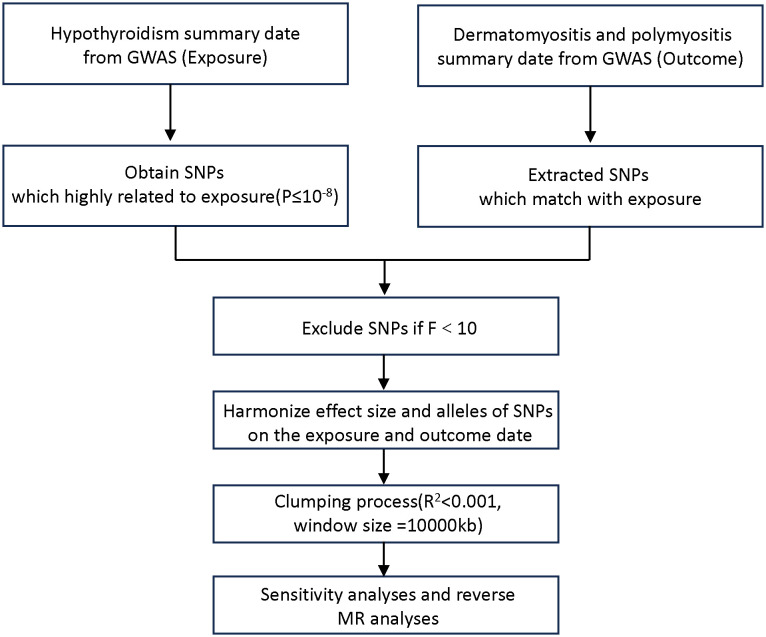
Flowchart of TSMR design.

The MR design investigates the causal relationship between a risk factor and an outcome based on observational data, using the random genetic assignment at conception as a natural experiment. To investigate the causal connections between hypothyroidism and DM/PM, in this TSMR analysis, we regard hypothyroidism as the exposure (risk factor) and DM/PM as the outcomes. We utilized data from the most recent genome-wide association studies (GWASs) on hypothyroidism and DM/PM obtained from the IEU Open GWAS database (https://gwas.mrcieu.ac.uk/datasets). The hypothyroidism GWAS summary dataset, which was used as the exposure, contained data for 410,141 individuals. There were two outcome datasets: polymyositis GWAS data (119 patients and 213,145 control individuals) and dermatomyositis GWAS data (201 patients and 172,834 control individuals). The disease diagnosis met the criteria of Phecode and ICD-10 (13, 14). **Figure 1** shows the flowchart of the MR study on the association between hypothyroidism and DM/PM.

A two-sample design necessitates that both samples represent similar underlying populations, with no overlap between them, as overlapping cases can introduce bias into MR estimates towards observed associations. Therefore, all participants in our two-sample MR study were drawn from distinct cohorts of European ancestry. Such selection typically enhances statistical power (11).

### 2.2 Instrumental variable selection

The selection of genetic instrumental variables is crucial for the success of Mendelian studies. To achieve unbiased estimation of the causal relationship between exposure and outcome, three core assumptions must be met (12): (1) associated with the risk factor; (2) not associated with any confounder of the risk factor outcome association; (3) not associated with the outcome conditional on the risk factor and confounders. To fulfil the assumptions, first, we selected the corresponding single-nucleotide polymorphisms (SNPs) from hypothyroidism exposure at the threshold of genome-wide significance (5×10^−8^). Then, to rule out a correlation with the outcome, linkage disequilibrium (LD) was estimated between SNPs to select independent genetic variants using the clump parameter (clumping window size = 10,000 kb, linkage disequilibrium coefficient R^2^ < 0.001) based on the 1000 genomes reference panel regarding Europeans as the superpopulation (15). Third, F statistics were calculated to estimate the sample overlap effect and weak instrument bias considering the relatively relaxed threshold, and an F < 10 was considered dubious bias (16).

Finally, to further address potential confounding factors, we utilized the PhenoScanner website (http://www.phenoscanner.medschl.cam.ac.uk/) to conduct additional screening of the selected instrumental variables. If any instrumental variable was found to be associated with risk factors related to the outcome, it was subsequently removed.

### 2.3 Pleiotropy and heterogeneity analysis

We used Cochran’s Q test to evaluate heterogeneity in the estimates of heterogeneity calculated by the inverse-variance weighting (IVW) and MR-Egger methods (17). Significant heterogeneity was indicated if P < 0.05, and a random-effects model was adopted in the subsequent analyses; otherwise, a fixed-effects model was adopted.

To ensure that instrumental variables did not influence DM or PM through other confounders or other biological pathways independent of hypothyroidism exposure, we performed the MR-Egger regression effects model and the MR pleiotropy residual sum and outlier test (MR-PRESSO) as pleiotropy test methods (18, 19) to check and correct directional pleiotropy. The effects of outlying IVs identified by MR-PRESSO tests were further evaluated in a distortion test, and any outliers associated with P < 0.05 were excluded and the causal estimates were reassessed. The MR Egger intercept test and P values were included in the analysis, and P > 0.05 indicated no significant pleiotropy. We also performed a “leave‐one‐out” analysis to investigate the possibility that the causal association was driven by a single SNP (20).

### 2.4 Statistical analyses

To evaluate the overall impact of hypothyroidism on DM/PM, we used the inverse variance weighted (IVW) as the main analysis method. When directional pleiotropy is absent, the IVW method can deliver a relatively stable and accurate causal evaluation by using a meta-analytic approach to combine Wald estimates for each instrumental variable. We also used MR-Egger, weighted median and weighted mode methods to evaluate the causal relationship between hypothyroidism exposure and DM/PM risk. MR-Egger allows for some of the SNPs to affect the outcome through mechanisms not involving modification of the exposure. Weighted median MR assumes that at least 50% of the SNPs are valid. Weighted-mode MR groups SNPs into clusters and calculates an estimate based on the cluster with the most SNPs. MR-Egger, weighted median, and weighted mode are alternative MR methods that are more robust to directional pleiotropy and were used to calculate estimates for comparison with the IVW estimates (21).

This TSMR analysis was performed using R software (version 4.3.1) with the TwoSampleMR (version 0.5.7) and MR-PRESSO packages (version 1.0.0). The significance threshold for instrumental variables that may have horizontal pleiotropy was P< 0.05, and P > 0.05 indicated no significant heterogeneity in the screened instrumental variables. Causal estimates were given as odds ratios (ORs) and 95% confidence intervals. We used publicly available summary data, so no ethical approval was needed.

## 3 Results

### 3.1 Extraction of instrumental variables of hypothyroidism from DM/PM GWAS datasets

After the instrument selection process described above, 68 SNPs were extracted from each of the two GWAS datasets, DM and PM. All of these SNPs were found to be associated with hypothyroidism at genome-wide significance (P< 5×10^−8^), making them potential instrumental variables for hypothyroidism and DM/PM (**Supplementary Table 1**). The F-statistics were all greater than 10, suggesting that weak instrument bias may not have been substantial. The effect alleles, other alleles, beta coefficients, standard errors (SE), and P-values of these instrumental variables were systematically collected for further analysis (**Supplementary Table 1**).

### 3.2 Two-sample MR analysis

**Figure 2:**
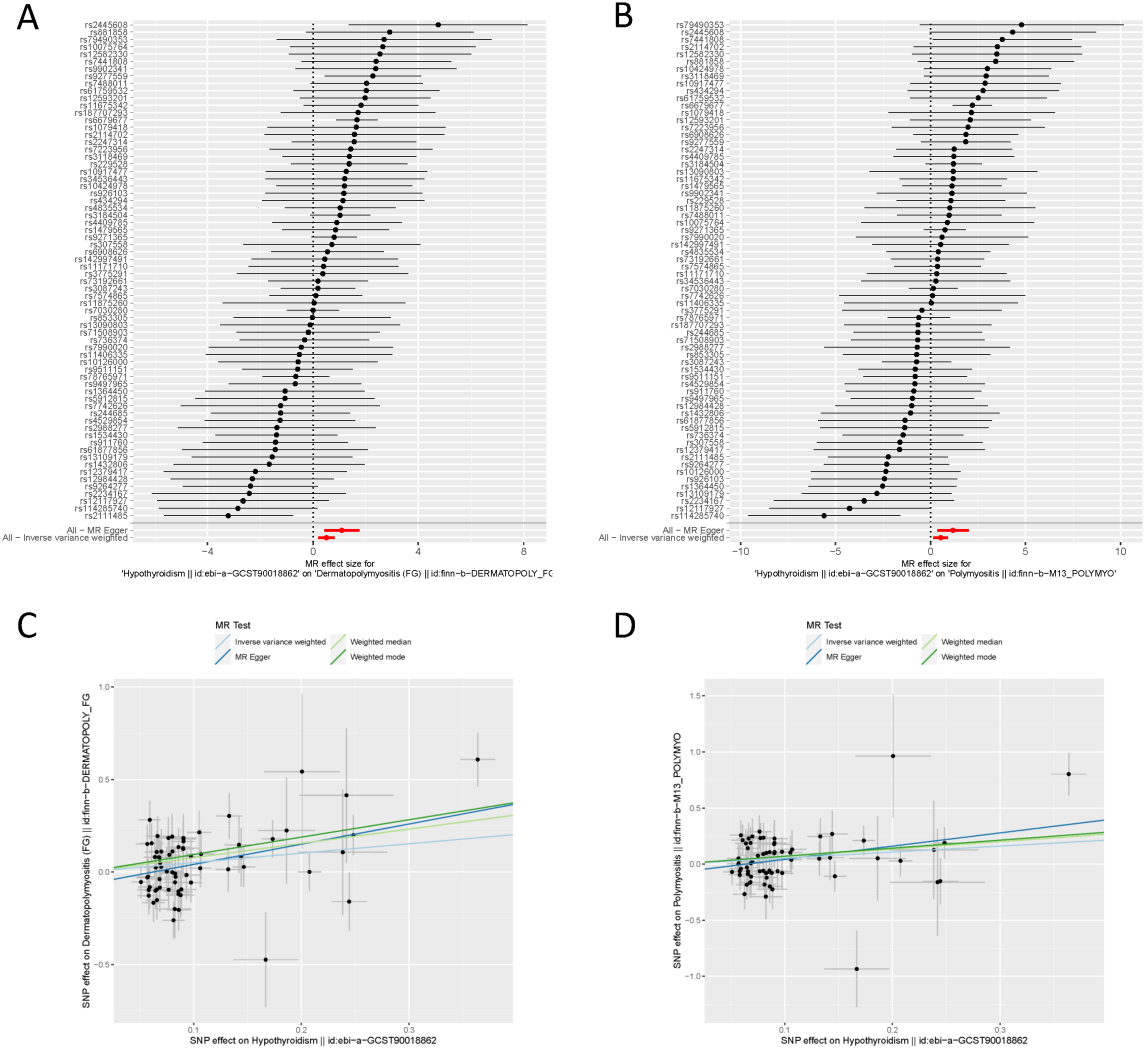
TSMR analysis of hypothyroidism and DM/PM. **(A)** Forest plot of hypothyroidism associated with risk of DM. All MR-Egger and IVM methods showed that MR effect sizes that are larger than 0 mean that hypothyroidism, drug reimbursement had a causal effect on DM. **(B)** Forest plot of hypothyroidism associated with risk of PM. All MR-Egger and IVM methods showed that MR effect sizes that are larger than 0 mean that hypothyroidism, drug reimbursement had a causal effect on PM. **(C)** MR test scatter plot of four methods. The x-axis is the SNP effect on hypothyroidism. The y-axis is the SNP effect on DM. **(D)** MR test scatter plot of four methods. The x-axis is the SNP effect on hypothyroidism. The y-axis is the SNP effect on PM. TSMR, two-sample mendelian randomization. DM, dermatomyositis. PM, polymyositis. SNP, single-nucleotide polymorphism.

**Figure 3:**
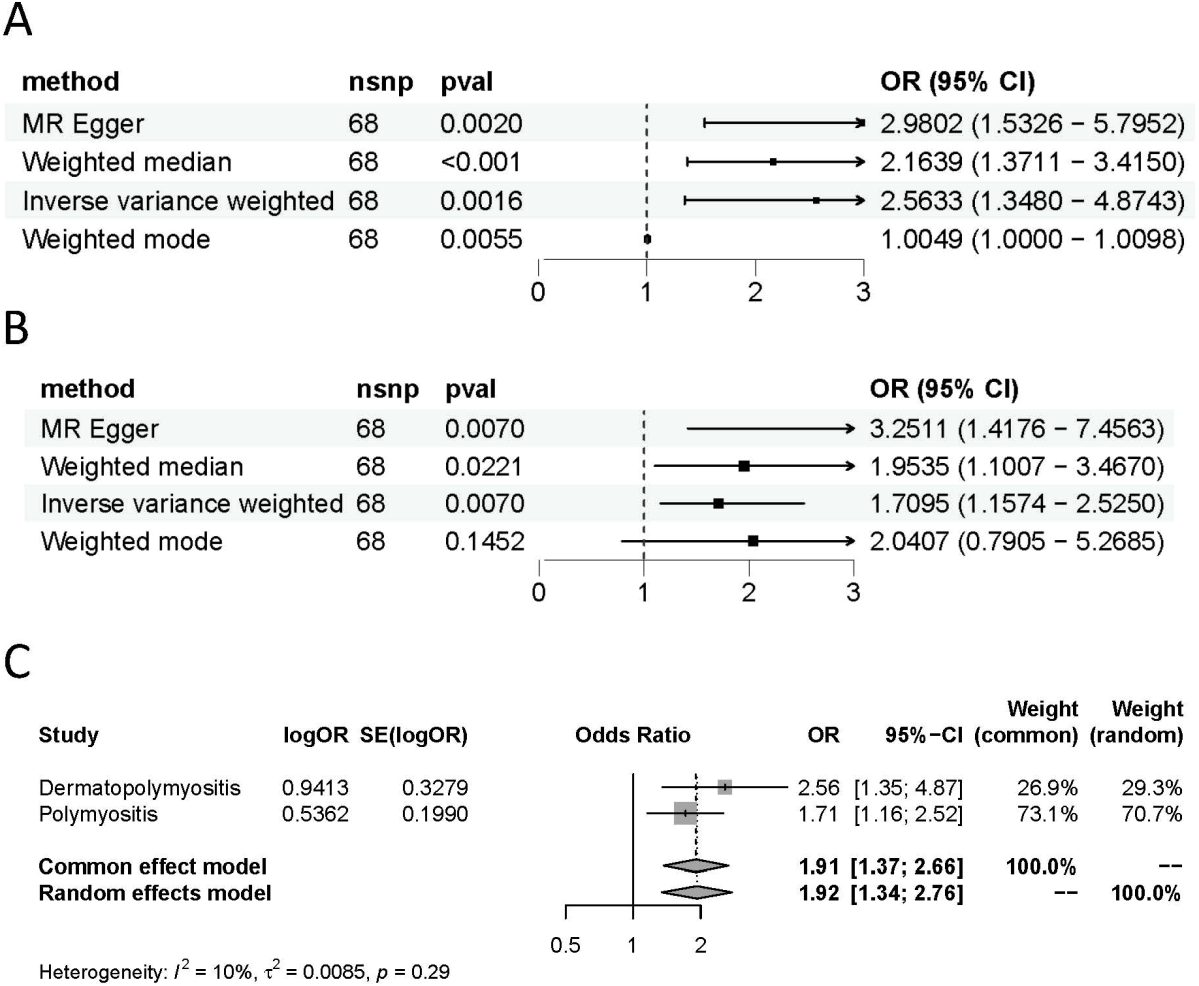
Causal estimates given as odds ratios (ORs) and 95% confidence intervals for the effect of hypothyroidism on DM **(A)** and PM **(B)**. **(C)** Meta-analysis of the results of the causal estimates between hypothyroidism on DM/PM, estimates were obtained from the IVW methods. DM, dermatomyositis. PM, polymyositis.

In the IVW component of our TSMR analysis, genetically predicted hypothyroidism was estimated to increase the risk of both DM and PM (OR = 2.563, 95% CI 1.348-4.874, P = 0.00156) and (OR =1.709, 95% CI 1.157-2.525, P = 0.007), respectively. The same results were shown in the other analysis methods (**Table 1**). As shown in **Table 1** and **Figures 2**, **3**, although the weighted mode of the PM GWAS MR analysis results was not significant, the results of the other three methods were significant, and the ORs of the four methods were all positive. **Figures 2A, B** show detailed forest plots with the estimated MR. Each solid horizontal line in the forest plot represents the results estimated based on a single SNP. The Wald ratio method was utilized, and the overall effect estimate (depicted by the red line at the bottom) is positioned entirely to the right of 0, indicating that increased exposure to hypothyroidism is associated with an increased risk of developing DM and PM.

**Table 1:** MR estimates from each method of assessing the causal effect of hypothyroidism on the risk of DM/PM.

Exposure	Outcome	No.SNP	Methods	β	SE	OR (95%CI)	Pvalue
Hypothyroidism	DM	68	MR Egger	1.092	0.339	2.980(1.53-5.80)	0.001994
			Weighted median	0.772	0.233	2.164(1.371-3.415)	0.000914
			Inverse variance weighted	0.508	0.161	2.563(1.348-4.874)	0.00156
			Weighted mode	0.941	0.328	1.005(1.000-1.001)	0.005473
Hypothyroidism	PM	68	MR Egger	1.179	0.423	3.251(1.418-7.456)	0.006997
			Weighted median	0.67	0.293	1.953(1.101-3.467)	0.02214
			Inverse variance weighted	0.536	0.199	1.709(1.157-2.525)	0.007049
			Weighted mode	0.713	0.484	2.041(0.790-5.268)	0.1452

DM, dermatomyositis; PM, polymyositis; SNP, single nucleotide polymorphism; β, beta coefficient; SE, standard error; OR, odds ratio. 95% CI, 95% confidence intervals. P <0.05 was considered statistically significant.

As demonstrated in **Table 1** and **Figures 2C, D**, each point in the scatter plot corresponds to a genetic variant (SNP), illustrating the association between that SNP and both exposure and outcome. Different lines represent the fits produced by different models, and slopes greater than 0 indicate that exposure to hypothyroidism is a risk factor for developing DM and PM. The regression lines obtained by these four methods were in the same direction, and the promoting effect of a single SNP on DM/PM increased as the effect of a single SNP on hypothyroidism increased. A summary view of the four methods all showed significant relationships between both hypothyroidism and DM/PM (**Figures 3A, B**). The results of TSMR prove that hypothyroidism is a risk factor for DM/PM at the genetic level.

To further clarify the overall impact of hypothyroidism on DM and PM, we performed a meta-analysis of the results of the main analytical method, IVW, which revealed an OR of 1.91 (95% CI 1.37-2.66), suggesting that hypothyroidism is a risk factor for DM and PM (**Figure 3C**). The results of the meta-analysis also validate the findings of TSMR. However, it is imperative to assess the heterogeneity and pleiotropy of the results. There was no evidence of heterogeneity (Q = 82.26, P = 0.0853) in Cochran’s Q test and no pleiotropy in the MR-Egger regression (intercept= -0.075, SE = 0.044, P= 0.0914) of the MR analysis of hypothyroidism and PM (**Table 2**).

**Table 2:** Pleiotropy and heterogeneity tests of hypothyroidism genetic instrumental variables in GWASs for DM/PM.

	Pleiotropy test			Heterogeneity test					
	MR Egger regression			Inverse variance weighted			MR Egger		
	intercept	SE	P	Q	Q_df	P	Q	Q_df	P
DM	-0.03768243	0.03628873	0.3030502	93.8865	66	0.289837	88.59461	67	0.2934329
PM	-0.075	0.044	0.0914	85.92	67	0.05959	82.26	66	0.0853

DM, dermatomyositis; PM, polymyositis; SE, standard error. P <0.05 can imply the presence of heterogeneity.

However, in the MR analysis of hypothyroidism and DM, significant heterogeneity (P_MR Egger_=0.03220071, P_IVW_= 0.01682226) was found via Cochran’s Q test. IV rs6679677 (RSSobs = 98.39573, p= 0.01333333) was identified as an outlier in the MR-PRESSO test and was excluded from the subsequent analyses, and no distortion was detected. After removing one outlier, there was no evidence of heterogeneity (P_MR Egger_=0.06065474, P_IVW_=0.06316350). No pleiotropy (intercept= -0.03768243, SE = 0.03628873, P= 0.3030502) was found in the MR analysis of hypothyroidism and DM (**Table 2**).

**Figure 4:**
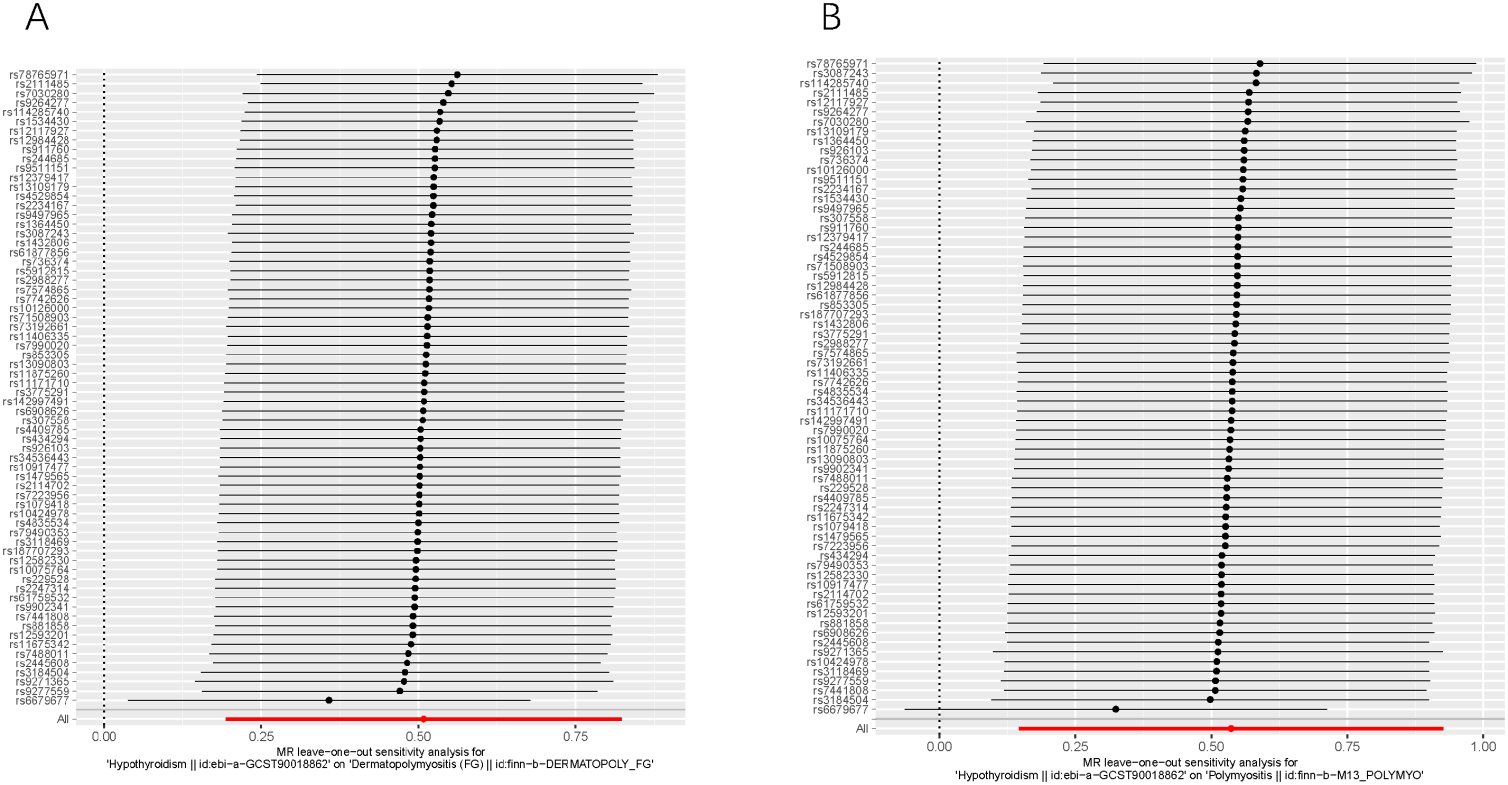
Leave-one-out of SNPs associated with hypothyroidism and their risk of DM **(A)** and PM **(B)**. Each black point represents result of the IVW MR method applied to estimate the causal effect of hypothyroidism on DM/PM. Each red point depicts the IVW estimate using all SNPs. No single SNP is strongly driving the overall effect of hypothyroidism on both DM and PM in this leave-one-out sensitivity analysis.

Additionally, as depicted in **Figure 4**, even after systematically removing individual SNPs in the leave-one-out sensitivity test, the effect of the MR result remained unchanged, and the overall effect direction remained consistent. This observation confirms the robustness of the result. Furthermore, funnel plots display symmetrical shapes, indicating the absence of pleiotropy in the causal relationships between genetically predicted hypothyroidism and the risk of DM/PM (**Supplementary Figure 1**).

Therefore, following the assessment for heterogeneity and pleiotropy, our results demonstrate that hypothyroidism exerts a significant causal effect on DM/PM and is a potential risk factor for DM/PM.

## 4 Discussion

This is the first study to detect the causal link between hypothyroidism and DM/PM using a TSMR method and to demonstrate that hypothyroidism increases the risk of DM/PM. Our study is based on European ancestry GWAS data, with a large population size, and the results of all four analyses showed a significant correlation between hypothyroidism and the risk of DM/PM. Our data support previous observational studies that have shown an association between hypothyroidism and DM/PM.

Idiopathic inflammatory myositis has been reported in association with various autoimmune and connective tissue diseases (22). However, there is relatively less information available regarding the coexistence of IIM and hypothyroidism. Some studies based on a small number of patients state that 5.5% of IIM patients were affected by dysthyroidism (23). The coexistence of hypothyroidism and poly/dermatomyositis was reported to be 11.2% and 25% in two different studies (8, 9).

The putative association of DM/PM with dysthyroidism was first reported in occasional case reports or case series since the 1960s, and most of those reports were based on small population studies (24–27). A retrospective study of 11 DM patients with autoimmune thyroiditis by WANG et al. suggested that autoimmune thyroiditis can precede or parallel the diagnosis of DM, and the most common comorbidities included hypothyroidism (90.9%). The most obvious is muscle damage, which was present in 100% of DM or PM patients with hypothyroidism, and the prevalence rates of abnormal myopathic manifestations were also found to be slightly higher than the data reported previously (28, 29). As reported previously, myalgia may occur in less than 30% of DM patients (22), but it occurs in 50% of PM patients with hypothyroidism (29). This significant difference directed our attention to the overlap between DM and hypothyroidism. Obviously, muscle tissue is the common target organ of DM and thyroid diseases; when DM or PM are combined with hypothyroidism, the probability of muscle injury increases significantly. Thyroid hormones are key regulators of the development, regeneration and metabolism of skeletal muscle (30). Triiodothyronine (T3), a bioactive thyroid hormone, affects muscle fiber contraction and relaxation by upregulating calcium fluxes and associated energy turnover (31). T3 has been found to correlate with the function of the sarcoplasmic reticulum calcineurin ATPase (SERCA) pump in myocytes (32). Hypothyroidism is characterized by muscle weakness caused by a decrease in muscle efficiency (33). Its symptoms may be more obvious due to superposition, which may affect the judgement of the efficacy of DM/PM. In addition, thyroid hormone is critical for the maintenance of cellular homeostasis during stress responses, which is implicated in affecting the apoptosis of structural and inflammatory cells as well as altering the cytokine microenvironment balance. Some studies have speculated that it probably plays an important role in the pathogenesis of pulmonary interstitial fibrosis and is often complicated with DM/PM (34, 35). Zhang et al. found that there was no response to standard immunosuppressive therapy in 75% of IIM patients and that correcting thyroid abnormalities could improve this situation. Thyroid dysfunction is also associated with the onset or recurrence of IIM (23).

Therefore, based on the above findings, we understand that there may be a biological link between hypothyroidism and DM, and the underlying mechanisms may be very complicated. In addition, hypothyroidism may contribute (directly or indirectly) to the development of PM/DM and may also exacerbate the clinical symptoms of DM and affect treatment outcomes.

In addition, it is worth noting that both DM and PM, especially anti-transcriptional intermediary factor 1-gamma antibody-positive dermatomyositis, are strongly associated with malignant diseases, such as ovarian, lung, pancreatic, enteron, and non-Hodgkin lymphoma, and the highest risk of these malignancies often occurs at the time of DM/PM diagnosis (36, 37). In Han Wang’s study of 17 DM/PM patients, 5 patients with malignant tumors were diagnosed with hypothyroidism (37). This suggests that hypothyroidism is not only a potential risk factor for human cancer (38) but should also be considered in the case of myositis.

However, due to the potential influence of confounding factors and reverse causality, it is difficult for conventional observational studies to establish the causal relationship between hypothyroidism and DM/PM, while the MR method is a promising tool for such an investigation since it uses genetic variations as instrumental variables of exposure factors to infer the causal relationship between exposure factors and outcomes (39). In our study, we confirmed the association of hypothyroidism with DM/PM at the genetic level and as a risk factor for DM/PM.

Although we observed directional pleiotropy in the analysis between hypothyroidism and DM in our study, it was adjusted by applying the MR-PRESSO test after excluding one potential outlier. The sensitivity test supported the stability and accuracy of the causal outcome. The results of our study provide evidence that the genetic risk of hypothyroidism is directly associated with PM/DM. It is an important reminder of the importance of preemptive endocrine hormone screening before disease onset and screening for hypothyroidism even after a DM diagnosis to alleviate the corresponding symptoms to avoid more serious damage to patients. However, the precise nature and direction of such a link need to be further explored, especially when designing prospective studies.

Although we obtained remarkable results from the TSMR approach, there are still some limitations in this study. First, the GWAS data of DM and PM were small, especially the number of patients. The larger the sample size of GWAS data, the stronger the statistical power. Second, data on myositis-specific antibodies in DM/PM patients were not available, making it impossible to categorize subsets and characteristics of thyroid dysfunction. Finally, due to the small number of studies, no comparative data have been obtained after hypothyroidism treatment, which indicates that more rigorous and comprehensive prospective studies are needed to further explore this relationship.

## 5 Conclusion

In conclusion, our results illustrate the significant causal effect of hypothyroidism on DM/PM. In addition, it is biologically plausible and explicable that hypothyroidism is important in the pathogenesis of DM/PM. Therefore, hypothyroidism should be considered and monitored closely. These findings add to a growing body of research aimed at identifying cures for DM/PM, and we have reasons to expect a breakthrough in the discovery of mechanisms and therapeutic targets for these conditions.

## Data Availability

The original contributions presented in the study are included in the article/**Supplementary Material**. Further inquiries can be directed to the corresponding author.

## Supplementary material

Supplementary Figure 1 MR funnel plot of IVW and MR-Egger methods DM **(A)** and PM **(B)**. IVW and MR‐Egger regression slopes were used to explore asymmetry as a sign of pleiotropy. DM, dermatomyositis. PM, polymyositis.

## References

[1] Lundberg IE Fujimoto M Vencovsky J Aggarwal R Holmqvist M Christopher-Stine L. Idiopathic inflammatory myopathies. Nat Rev Dis primers. (2021) 7:86. doi: 10.1038/s41572-021-00321-x 34857798

[2] Dalakas MC. Inflammatory muscle diseases. New Engl J Med. (2015) 372:1734–47. doi: 10.1056/NEJMra1402225 25923553

[3] Lilleker JB Vencovsky J Wang G Wedderburn LR Diederichsen LP Schmidt J. The EuroMyositis registry: an international collaborative tool to facilitate myositis research. Ann rheumatic diseases. (2018) 77:30–9. doi: 10.1136/annrheumdis-2017-211868 PMC5754739 28855174

[4] Miller FW Lamb JA Schmidt J Nagaraju K. Risk factors and disease mechanisms in myositis. Nat Rev Rheumatol. (2018) 14:255–68. doi: 10.1038/nrrheum.2018.48 PMC6745704 29674613

[5] Dalakas MC. Inflammatory muscle diseases: a critical review on pathogenesis and therapies. Curr Opin Pharmacol. (2010) 10:346–52. doi: 10.1016/j.coph.2010.03.001 20409756

[6] Hollowell JG Staehling NW Flanders WD Hannon WH Gunter EW Spencer CA. T(4), and thyroid antibodies in the United States population (1988 to 1994): National Health and Nutrition Examination Survey (NHANES III). J Clin Endocrinol Metab. (2002) 87:489–99. doi: 10.1210/jcem.87.2.8182 11836274

[7] Mahagna H Caplan A Watad A Bragazzi NL Sharif K Tiosano S. Rheumatoid arthritis and thyroid dysfunction: A cross-sectional study and a review of the literature. Best Pract Res Clin Rheumatol. (2018) 32:683–91. doi: 10.1016/j.berh.2019.01.021 31203926

[8] Lukjanowicz M Bobrowska-Snarska D Brzosko M. Coexistence of hypothyroidism with polymyositis or dermatomyositis. Annales Academiae Medicae Stetinensis. (2006) 52 Suppl 2:49–55. 17471837

[9] Watad A Bragazzi NL Damiani G Nissan E Comaneshter D Cohen AD. Dysthyroidism in dermato/polymyositis patients: A case-control study. Eur J Clin Invest. (2021) 51:e13460. doi: 10.1111/eci.13460 33283286

[10] Burgess S Daniel RM Butterworth AS Thompson SG. Network Mendelian randomization: using genetic variants as instrumental variables to investigate mediation in causal pathways. Int J Epidemiol. (2015) 44:484–95. doi: 10.1093/ije/dyu176 PMC4469795 25150977

[11] Larsson SC Butterworth AS Burgess S. Mendelian randomization for cardiovascular diseases: principles and applications. Eur Heart J. (2023) 44:4913–24. doi: 10.1093/eurheartj/ehad736 PMC10719501 37935836

[12] Davey Smith G Hemani G. Mendelian randomization: genetic anchors for causal inference in epidemiological studies. Hum Mol Genet. (2014) 23:R89–98. doi: 10.1093/hmg/ddu328 PMC4170722 25064373

[13] Denny JC Bastarache L Ritchie MD Carroll RJ Zink R Mosley JD. Systematic comparison of phenome-wide association study of electronic medical record data and genome-wide association study data. Nat Biotechnol. (2013) 31:1102–10. doi: 10.1038/nbt.2749 PMC3969265 24270849

[14] Organización Mundial de la Salud. International Statistical Classification of Diseases and Related Health Problems, 10th revision. (ICD-10) (World Health 2016).

[15] Richardson TG Sanderson E Elsworth B Tilling K Davey Smith G. Use of genetic variation to separate the effects of early and later life adiposity on disease risk: mendelian randomisation study. BMJ (Clinical Res ed). (2020) 369:m1203. doi: 10.1136/bmj.m1203 PMC7201936 32376654

[16] Burgess S Thompson SG. Bias in causal estimates from Mendelian randomization studies with weak instruments. Stat Med. (2011) 30:1312–23. doi: 10.1002/sim.4197 21432888

[17] Bowden J Hemani G Davey Smith G. Invited commentary: detecting individual and global horizontal pleiotropy in mendelian randomization-A job for the humble heterogeneity statistic? Am J Epidemiol. (2018) 187:2681–5. doi: 10.1093/aje/kwy185 PMC6269239 30188969

[18] Burgess S Thompson SG. Interpreting findings from Mendelian randomization using the MR-Egger method. Eur J Epidemiol. (2017) 32:377–89. doi: 10.1007/s10654-017-0255-x PMC5506233 28527048

[19] Verbanck M Chen CY Neale B Do R. Detection of widespread horizontal pleiotropy in causal relationships inferred from Mendelian randomization between complex traits and diseases. Nat Genet. (2018) 50:693–8. doi: 10.1038/s41588-018-0099-7 PMC6083837 29686387

[20] Lee YH Song GG. Uric acid level, gout and bone mineral density: A Mendelian randomization study. Eur J Clin Invest. (2019) 49:e13156. doi: 10.1111/eci.13156 31294819

[21] Hemani G Bowden J Davey Smith G. Evaluating the potential role of pleiotropy in Mendelian randomization studies. Hum Mol Genet. (2018) 27:R195–r208. doi: 10.1093/hmg/ddy163 29771313 PMC6061876

[22] Dalakas MC Hohlfeld R. Polymyositis and dermatomyositis. Lancet (London England). (2003) 362:971–82. doi: 10.1016/S0140-6736(03)14368-1 14511932

[23] Selva-O’Callaghan A Redondo-Benito A Trallero-Araguás E Martínez-Gómez X Palou E Vilardell-Tarres M. Clinical significance of thyroid disease in patients with inflammatory myopathy. Medicine. (2007) 86:293–8. doi: 10.1097/MD.0b013e318156f9c2 17873759

[24] Nagy E Szodoray L Pongracz E. Simultaneous presence of Hashimoto’s goiter, dermatomyositis and scleromyxedema. Z fur Haut- und Geschlechtskrankheiten. (1962) 33:26–9. 14477973

[25] Gamsky TE Chan MK. Coexistent dermatomyositis and autoimmune thyroiditis. Western J Med. (1988) 148:213–4. PMC1026074 3348033

[26] Go T Mitsuyoshi I. Juvenile dermatomyositis associated with subclinical hypothyroidism due to auto-immune thyroiditis. Eur J pediatrics. (2002) 161:358–9. doi: 10.1007/s00431-002-0947-3 12029461

[27] Charalabopoulos K Mittari E Peschos D Golias C Charalabopoulos A Tsanou E. Rare association of chronic lymphocytic thyroiditis with dermatomyositis. Arch Med Res. (2006) 37:563–5. doi: 10.1016/j.arcmed.2005.11.001 16624661

[28] Wang H Tao L Li H Deng J. Dermatomyositis related to autoimmune thyroiditis. J Eur Acad Dermatol Venereology: JEADV. (2011) 25:1085–93. doi: 10.1111/j.1468-3083.2010.03929.x 21118310

[29] Wang H Li H Kai C Deng J. Polymyositis associated with hypothyroidism or hyperthyroidism: two cases and review of the literature. Clin Rheumatol. (2011) 30:449–58. doi: 10.1007/s10067-010-1570-8 20857158

[30] Bloise FF Cordeiro A Ortiga-Carvalho TM. Role of thyroid hormone in skeletal muscle physiology. J Endocrinol. (2018) 236:R57–r68. doi: 10.1530/JOE-16-0611 29051191

[31] Kiss E Jakab G Kranias EG Edes I. Thyroid hormone-induced alterations in phospholamban protein expression. Regulatory effects on sarcoplasmic reticulum Ca2+ transport and myocardial relaxation. Circ Res. (1994) 75:245–51. doi: 10.1161/01.RES.75.2.245 8033338

[32] Simonides WS Thelen MH van der Linden CG Muller A van Hardeveld C. Mechanism of thyroid-hormone regulated expression of the SERCA genes in skeletal muscle: implications for thermogenesis. Bioscience Rep. (2001) 21:139–54. doi: 10.1023/A:1013692023449 11725863

[33] Zürcher RM Horber FF Grünig BE Frey FJ. Effect of thyroid dysfunction on thigh muscle efficiency. J Clin Endocrinol Metab. (1989) 69:1082–6. doi: 10.1210/jcem-69-5-1082 2793992

[34] Fois AG Paliogiannis P Sotgia S Mangoni AA Zinellu E Pirina P. Evaluation of oxidative stress biomarkers in idiopathic pulmonary fibrosis and therapeutic applications: a systematic review. Respir Res. (2018) 19:51. doi: 10.1186/s12931-018-0754-7 29587761 PMC5872514

[35] Mastruzzo C Crimi N Vancheri C. Role of oxidative stress in pulmonary fibrosis. Monaldi Arch chest Dis = Archivio Monaldi per le malattie del torace. (2002) 57:173–6. 12619377

[36] Hill CL Zhang Y Sigurgeirsson B Pukkala E Mellemkjaer L Airio A. Frequency of specific cancer types in dermatomyositis and polymyositis: a population-based study. Lancet (London England). (2001) 357:96–100. doi: 10.1016/S0140-6736(00)03540-6 11197446

[37] Zampieri S Valente M Adami N Biral D Ghirardello A Rampudda ME. Polymyositis, dermatomyositis and Malignancy: a further intriguing link. Autoimmun Rev. (2010) 9:449–53. doi: 10.1016/j.autrev.2009.12.005 20026430

[38] Kuijpens JL Nyklíctek I Louwman MW Weetman TA Pop VJ Coebergh JW. Hypothyroidism might be related to breast cancer in post-menopausal women. Thyroid: Off J Am Thyroid Assoc. (2005) 15:1253–9. doi: 10.1089/thy.2005.15.1253 16356089

[39] Dong SS Zhang K Guo Y Ding JM Rong Y Feng JC. Phenome-wide investigation of the causal associations between childhood BMI and adult trait outcomes: a two-sample Mendelian randomization study. Genome Med. (2021) 13:48. doi: 10.1186/s13073-021-00865-3 33771188 PMC8004431

