# Supplementary figures and images for: Hypothyroidism and dermato/polymyositis: a two-sample Mendelian randomization study
Source: Front Endocrinol (Lausanne). 2024 Sep 4;15:1361581. doi: 10.3389/fendo.2024.1361581 (PMC11408279; doi:10.3389/fendo.2024.1361581)

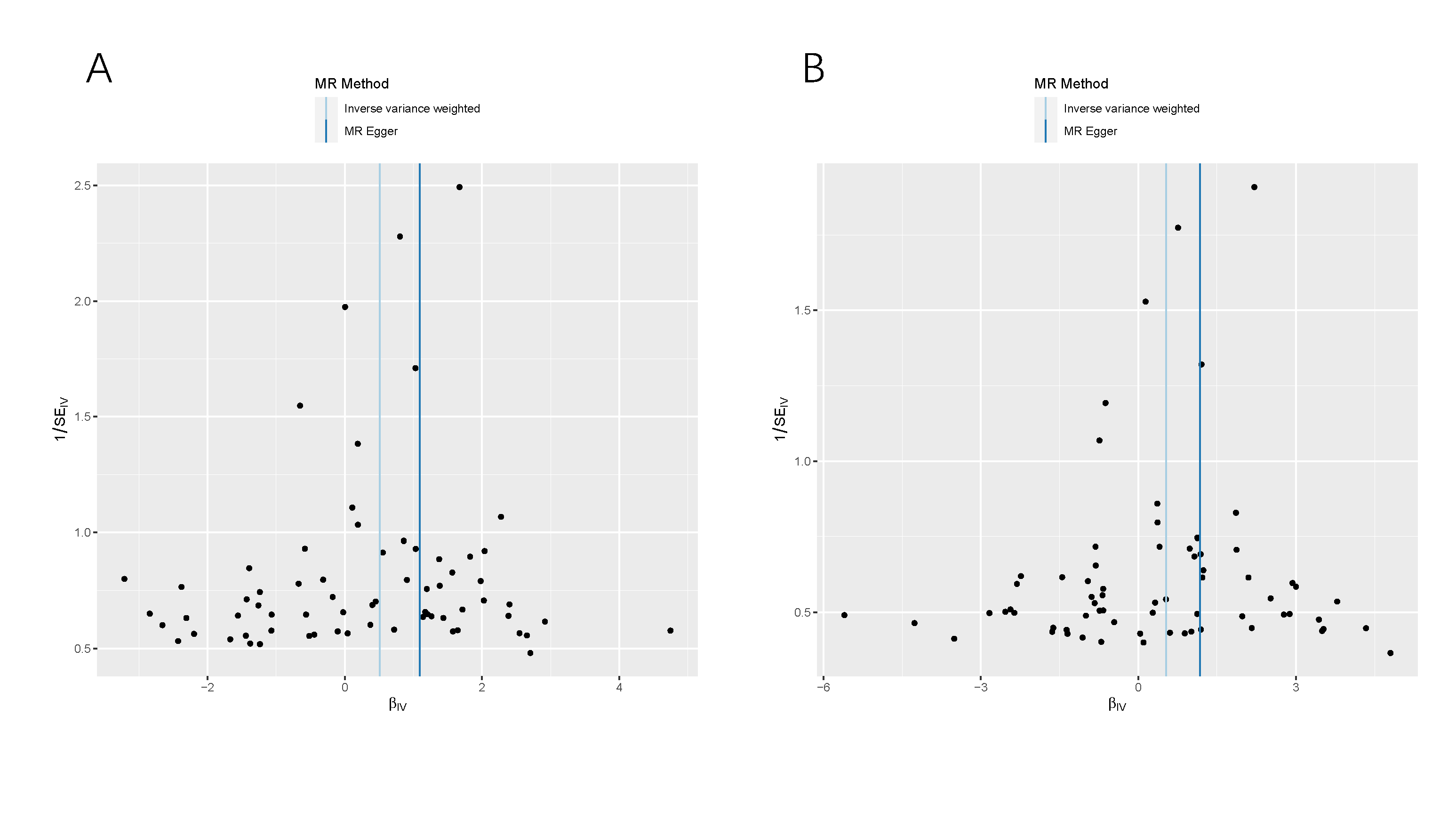

Supplement: Supplementary Figure 1 — MR funnel plot of IVW and MR-Egger methods DM (A) and PM (B). IVW and MR‐Egger regression slopes were used to explore asymmetry as a sign of pleiotropy. DM, dermatomyositis. PM, polymyositis. [file Image1.tif]
