# Supplementary material for: Hypothyroidism and dermato/polymyositis: a two-sample Mendelian randomization study
Source: Front Endocrinol (Lausanne). 2024 Sep 4;15:1361581. doi: 10.3389/fendo.2024.1361581 (PMC11408279; doi:10.3389/fendo.2024.1361581)
Supplement: Supplementary file 2 [file Table1.docx]

| **Supplementary Table 1. Genetic variants used as potential instruments in the Mendelian randomization analyses investigating the effects of hypothyroidism on DM/PM.** | | | | | | | | | | | | | | |
| --- | --- | --- | --- | --- | --- | --- | --- | --- | --- | --- | --- | --- | --- | --- |
| **Exposure** | **Outcome** | **SNP** | **CHR** | **POS** | **EA_exp** | **OA_exp** | **EA_otcm** | **OA_otcm** | **b_exp** | **se_exp** | **pval_exp** | **b_otcm** | **se_otcm** | **pval_otcm** |
| Hypothyroidism | Dermatopolymyositis | rs10075764 | 5 | 35841449 | G | A | G | A | -0.057 | 0.0104 | 4.26E-08 | -0.1513 | 0.1024 | 0.1395 |
| Hypothyroidism | Dermatopolymyositis | rs10126000 | 9 | 21578617 | A | C | A | C | -0.0683 | 0.0104 | 5.13E-11 | 0.0388 | 0.1056 | 0.7134 |
| Hypothyroidism | Dermatopolymyositis | rs10424978 | 19 | 4837557 | A | C | A | C | -0.0775 | 0.0102 | 2.77E-14 | -0.0925 | 0.1023 | 0.3655 |
| Hypothyroidism | Dermatopolymyositis | rs1079418 | 6 | 1.66E+08 | G | A | G | A | -0.0657 | 0.011 | 2.14E-09 | -0.108 | 0.1135 | 0.3413 |
| Hypothyroidism | Dermatopolymyositis | rs10917477 | 1 | 19861106 | G | A | G | A | 0.064 | 0.01 | 1.75E-10 | 0.0808 | 0.1001 | 0.4196 |
| Hypothyroidism | Dermatopolymyositis | rs11171710 | 12 | 56368078 | A | G | A | G | -0.0698 | 0.01 | 3.19E-12 | -0.0279 | 0.1014 | 0.7833 |
| Hypothyroidism | Dermatopolymyositis | rs11406335 | 13 | 99934479 | TG | T | TG | T | -0.057 | 0.0103 | 3.44E-08 | 0.0297 | 0.1028 | 0.772899 |
| Hypothyroidism | Dermatopolymyositis | rs114285740 | 1 | 1.08E+08 | C | G | C | G | 0.1669 | 0.0301 | 3.06E-08 | -0.4743 | 0.2565 | 0.06442 |
| Hypothyroidism | Dermatopolymyositis | rs11675342 | 2 | 1407628 | T | C | T | C | 0.0906 | 0.01 | 1.4E-19 | 0.1654 | 0.101 | 0.1016 |
| Hypothyroidism | Dermatopolymyositis | rs11875260 | 18 | 77179239 | G | A | G | A | 0.0751 | 0.0135 | 2.54E-08 | 0.0029 | 0.1328 | 0.9825 |
| Hypothyroidism | Dermatopolymyositis | rs12117927 | 1 | 2.37E+08 | A | C | A | C | 0.0627 | 0.0105 | 2.29E-09 | -0.1666 | 0.1043 | 0.1104 |
| Hypothyroidism | Dermatopolymyositis | rs12379417 | 9 | 1.27E+08 | A | G | A | G | 0.0583 | 0.0103 | 1.51E-08 | -0.128 | 0.1035 | 0.2162 |
| Hypothyroidism | Dermatopolymyositis | rs12582330 | 12 | 1.04E+08 | T | G | T | G | -0.061 | 0.0109 | 2.05E-08 | -0.1553 | 0.1077 | 0.1491 |
| Hypothyroidism | Dermatopolymyositis | rs12593201 | 15 | 38844106 | A | G | A | G | 0.0905 | 0.0112 | 7.69E-16 | 0.1792 | 0.1143 | 0.1167 |
| Hypothyroidism | Dermatopolymyositis | rs12984428 | 19 | 50199616 | A | G | A | G | -0.0659 | 0.0102 | 1.11E-10 | 0.1521 | 0.1043 | 0.1446 |
| Hypothyroidism | Dermatopolymyositis | rs13090803 | 3 | 1.06E+08 | T | G | T | G | 0.0829 | 0.0128 | 9E-11 | -0.0088 | 0.1444 | 0.9515 |
| Hypothyroidism | Dermatopolymyositis | rs13109179 | 4 | 10727528 | A | G | A | G | 0.0647 | 0.01 | 9.42E-11 | -0.1006 | 0.1007 | 0.3179 |
| Hypothyroidism | Dermatopolymyositis | rs1364450 | 6 | 1.09E+08 | C | A | C | A | 0.0886 | 0.0139 | 1.97E-10 | -0.0942 | 0.137 | 0.4917 |
| Hypothyroidism | Dermatopolymyositis | rs142997491 | 16 | 50729820 | G | A | G | A | 0.2385 | 0.0412 | 7.02E-09 | 0.1067 | 0.3392 | 0.753101 |
| Hypothyroidism | Dermatopolymyositis | rs1432806 | 5 | 1.09E+08 | G | A | G | A | 0.0583 | 0.0105 | 2.89E-08 | -0.0972 | 0.108 | 0.368 |
| Hypothyroidism | Dermatopolymyositis | rs1479565 | 5 | 76544157 | A | G | A | G | 0.0975 | 0.0101 | 7.53E-22 | 0.0836 | 0.1011 | 0.4084 |
| Hypothyroidism | Dermatopolymyositis | rs1534430 | 2 | 12644736 | T | C | T | C | -0.086 | 0.0101 | 1.44E-17 | 0.1196 | 0.1015 | 0.2384 |
| Hypothyroidism | Dermatopolymyositis | rs187707293 | 4 | 1.8E+08 | A | T | A | T | 0.2419 | 0.044 | 3.99E-08 | 0.4146 | 0.3619 | 0.2519 |
| Hypothyroidism | Dermatopolymyositis | rs2111485 | 2 | 1.63E+08 | G | A | G | A | 0.0813 | 0.0102 | 1.43E-15 | -0.2611 | 0.1015 | 0.01012 |
| Hypothyroidism | Dermatopolymyositis | rs2114702 | 14 | 81405135 | A | T | A | T | 0.07 | 0.0111 | 3E-10 | 0.1103 | 0.1219 | 0.3654 |
| Hypothyroidism | Dermatopolymyositis | rs2234167 | 1 | 2494330 | A | G | A | G | 0.0825 | 0.015 | 3.75E-08 | -0.2003 | 0.155 | 0.1962 |
| Hypothyroidism | Dermatopolymyositis | rs2247314 | 6 | 1.67E+08 | C | T | C | T | -0.086 | 0.0104 | 1.06E-16 | -0.1346 | 0.1038 | 0.1949 |
| Hypothyroidism | Dermatopolymyositis | rs229528 | 22 | 37581677 | T | C | T | C | 0.0903 | 0.01 | 2.31E-19 | 0.124 | 0.1019 | 0.2239 |
| Hypothyroidism | Dermatopolymyositis | rs2412976 | 22 | 30567907 | G | C | G | C | 0.0637 | 0.0103 | 5.47E-10 | 0.086 | 0.1072 | 0.4227 |
| Hypothyroidism | Dermatopolymyositis | rs2445608 | 8 | 1.28E+08 | A | G | A | G | -0.0593 | 0.0101 | 3.79E-09 | -0.2816 | 0.1027 | 0.006119 |
| Hypothyroidism | Dermatopolymyositis | rs244685 | 5 | 1.33E+08 | G | T | G | T | -0.0858 | 0.0132 | 7.06E-11 | 0.106 | 0.1153 | 0.3579 |
| Hypothyroidism | Dermatopolymyositis | rs2921053 | 8 | 8319963 | C | G | C | G | -0.0599 | 0.0101 | 3.36E-09 | -0.0551 | 0.1794 | 0.7586 |
| Hypothyroidism | Dermatopolymyositis | rs2988277 | 1 | 1.67E+08 | T | C | T | C | 0.0593 | 0.0106 | 2.49E-08 | -0.0816 | 0.1137 | 0.472799 |
| Hypothyroidism | Dermatopolymyositis | rs307558 | 3 | 12095130 | A | G | A | G | -0.0688 | 0.0119 | 8.01E-09 | -0.0493 | 0.1183 | 0.676901 |
| Hypothyroidism | Dermatopolymyositis | rs3087243 | 2 | 2.05E+08 | A | G | A | G | -0.1466 | 0.0102 | 4.77E-47 | -0.0273 | 0.106 | 0.7969 |
| Hypothyroidism | Dermatopolymyositis | rs3118469 | 10 | 6101129 | T | A | T | A | 0.0803 | 0.0106 | 3.82E-14 | 0.111 | 0.1041 | 0.286 |
| Hypothyroidism | Dermatopolymyositis | rs3184504 | 12 | 1.12E+08 | C | T | C | T | -0.1734 | 0.0102 | 7.5E-65 | -0.1783 | 0.1014 | 0.078619 |
| Hypothyroidism | Dermatopolymyositis | rs34536443 | 19 | 10463118 | C | G | C | G | -0.1863 | 0.0263 | 1.46E-12 | -0.2241 | 0.2871 | 0.4351 |
| Hypothyroidism | Dermatopolymyositis | rs3775291 | 4 | 1.87E+08 | T | C | T | C | -0.0649 | 0.0108 | 1.65E-09 | -0.024 | 0.1077 | 0.8238 |
| Hypothyroidism | Dermatopolymyositis | rs434294 | 5 | 1.03E+08 | G | A | G | A | -0.0683 | 0.0109 | 3.36E-10 | -0.0778 | 0.1073 | 0.4684 |
| Hypothyroidism | Dermatopolymyositis | rs4409785 | 11 | 95311422 | C | T | C | T | 0.1069 | 0.0133 | 8.04E-16 | 0.0966 | 0.1342 | 0.4717 |
| Hypothyroidism | Dermatopolymyositis | rs4529854 | 10 | 64043975 | T | C | T | C | -0.0768 | 0.0107 | 6.56E-13 | 0.0965 | 0.1119 | 0.3885 |
| Hypothyroidism | Dermatopolymyositis | rs4835534 | 4 | 1.5E+08 | C | T | C | T | -0.1421 | 0.0132 | 7.06E-27 | -0.1466 | 0.1527 | 0.3371 |
| Hypothyroidism | Dermatopolymyositis | rs5912815 | X | 78466147 | G | T | G | T | -0.0511 | 0.0084 | 1.05E-09 | 0.0545 | 0.0885 | 0.5382 |
| Hypothyroidism | Dermatopolymyositis | rs61759532 | 17 | 7240391 | T | C | T | C | 0.0905 | 0.0122 | 1.42E-13 | 0.1833 | 0.1279 | 0.1518 |
| Hypothyroidism | Dermatopolymyositis | rs61877856 | 11 | 617378 | T | C | T | C | -0.0658 | 0.0115 | 1.14E-08 | 0.0947 | 0.1185 | 0.4241 |
| Hypothyroidism | Dermatopolymyositis | rs6679677 | 1 | 1.14E+08 | A | C | A | C | 0.3637 | 0.0159 | 2.4E-115 | 0.6078 | 0.1459 | 3.12E-05 |
| Hypothyroidism | Dermatopolymyositis | rs6908626 | 6 | 91005743 | T | G | T | G | 0.1441 | 0.0141 | 2.04E-24 | 0.0795 | 0.1575 | 0.6136 |
| Hypothyroidism | Dermatopolymyositis | rs7030280 | 9 | 1.01E+08 | T | C | T | C | 0.2075 | 0.0108 | 1.02E-82 | 0.0003 | 0.1051 | 0.9975 |
| Hypothyroidism | Dermatopolymyositis | rs71508903 | 10 | 63779871 | T | C | T | C | 0.0934 | 0.0125 | 9.34E-14 | -0.0168 | 0.1292 | 0.8963 |
| Hypothyroidism | Dermatopolymyositis | rs7223956 | 17 | 45373844 | C | T | C | T | -0.0902 | 0.0144 | 4.27E-10 | -0.1294 | 0.1427 | 0.3646 |
| Hypothyroidism | Dermatopolymyositis | rs73192661 | 3 | 1.88E+08 | T | C | T | C | -0.1061 | 0.01 | 4.05E-26 | -0.02 | 0.1027 | 0.8454 |
| Hypothyroidism | Dermatopolymyositis | rs736374 | 11 | 35266944 | A | G | A | G | 0.0832 | 0.0103 | 6E-16 | -0.0265 | 0.1043 | 0.7996 |
| Hypothyroidism | Dermatopolymyositis | rs7441808 | 4 | 26090375 | G | A | G | A | 0.0766 | 0.0111 | 5.17E-12 | 0.1838 | 0.1109 | 0.09734 |
| Hypothyroidism | Dermatopolymyositis | rs7488011 | 12 | 9925336 | T | C | T | C | 0.1052 | 0.0111 | 2.52E-21 | 0.2143 | 0.1142 | 0.06066 |
| Hypothyroidism | Dermatopolymyositis | rs7574865 | 2 | 1.92E+08 | G | T | G | T | -0.1321 | 0.0117 | 1.67E-29 | -0.0141 | 0.1193 | 0.9061 |
| Hypothyroidism | Dermatopolymyositis | rs7742626 | 6 | 1.36E+08 | C | T | C | T | 0.0686 | 0.0116 | 3.41E-09 | -0.0847 | 0.1322 | 0.5217 |
| Hypothyroidism | Dermatopolymyositis | rs78765971 | 1 | 1.08E+08 | G | C | G | C | 0.2444 | 0.0162 | 1.68E-51 | -0.1602 | 0.1579 | 0.3102 |
| Hypothyroidism | Dermatopolymyositis | rs79490353 | 13 | 28623048 | C | T | C | T | 0.2006 | 0.0349 | 8.82E-09 | 0.5425 | 0.4174 | 0.1937 |
| Hypothyroidism | Dermatopolymyositis | rs7990020 | 13 | 43044946 | C | A | C | A | 0.0577 | 0.0101 | 9.97E-09 | -0.0259 | 0.103 | 0.8014 |
| Hypothyroidism | Dermatopolymyositis | rs853305 | 8 | 1.34E+08 | C | T | C | T | -0.0802 | 0.0111 | 4.4E-13 | 0.0021 | 0.1221 | 0.9864 |
| Hypothyroidism | Dermatopolymyositis | rs881858 | 6 | 43806609 | A | G | A | G | 0.0665 | 0.0108 | 8.46E-10 | 0.194 | 0.1079 | 0.07221 |
| Hypothyroidism | Dermatopolymyositis | rs911760 | 9 | 5438435 | A | C | A | C | 0.0879 | 0.0125 | 1.95E-12 | -0.1255 | 0.1233 | 0.3087 |
| Hypothyroidism | Dermatopolymyositis | rs926103 | 1 | 1.57E+08 | C | T | C | T | -0.0678 | 0.0104 | 7.65E-11 | -0.0794 | 0.103 | 0.4409 |
| Hypothyroidism | Dermatopolymyositis | rs9264277 | 6 | 31224667 | C | T | C | T | -0.0862 | 0.0111 | 9.03E-15 | 0.205 | 0.1125 | 0.06838 |
| Hypothyroidism | Dermatopolymyositis | rs9271365 | 6 | 32586794 | G | T | G | T | 0.2484 | 0.0105 | 4.9E-123 | 0.1993 | 0.109 | 0.067569 |
| Hypothyroidism | Dermatopolymyositis | rs9277559 | 6 | 33056731 | C | T | C | T | -0.133 | 0.012 | 1.86E-28 | -0.3028 | 0.1246 | 0.01508 |
| Hypothyroidism | Dermatopolymyositis | rs9497965 | 6 | 1.49E+08 | T | C | T | C | 0.0827 | 0.0102 | 3.71E-16 | -0.056 | 0.106 | 0.5971 |
| Hypothyroidism | Dermatopolymyositis | rs9511151 | 13 | 24786576 | A | G | A | G | -0.0976 | 0.0106 | 3.08E-20 | 0.0571 | 0.1048 | 0.5856 |
| Hypothyroidism | Dermatopolymyositis | rs9902341 | 17 | 40278522 | T | C | T | C | 0.0801 | 0.0129 | 4.68E-10 | 0.1911 | 0.1249 | 0.1261 |
| Hypothyroidism | Dermatopolymyositis | rs3129967 | 6 | 32381461 | T | C | T | C | 0.3826 | 0.0577 | 3.45E-11 | 0.4312 | 0.1188 | 0.000283 |
| Hypothyroidism | Dermatopolymyositis | rs9295987 | 6 | 31349844 | G | A | G | A | 1.0568 | 0.1048 | 6.71E-24 | 0.248 | 0.2277 | 0.2761 |
| Hypothyroidism | Polymyositis | rs10075764 | 5 | 35841449 | G | A | G | A | -0.057 | 0.0104 | 4.26E-08 | 0.7022 | 0.1325 | 0.7022 |
| Hypothyroidism | Polymyositis | rs10126000 | 9 | 21578617 | A | C | A | C | -0.0683 | 0.0104 | 5.13E-11 | 0.2399 | 0.1371 | 0.2399 |
| Hypothyroidism | Polymyositis | rs10424978 | 19 | 4837557 | A | C | A | C | -0.0775 | 0.0102 | 2.77E-14 | 0.07931 | 0.1325 | 0.07931 |
| Hypothyroidism | Polymyositis | rs1079418 | 6 | 1.66E+08 | G | A | G | A | -0.0657 | 0.011 | 2.14E-09 | 0.3341 | 0.1467 | 0.3341 |
| Hypothyroidism | Polymyositis | rs10917477 | 1 | 19861106 | G | A | G | A | 0.064 | 0.01 | 1.75E-10 | 0.1546 | 0.1295 | 0.1546 |
| Hypothyroidism | Polymyositis | rs11171710 | 12 | 56368078 | A | G | A | G | -0.0698 | 0.01 | 3.19E-12 | 0.8633 | 0.1312 | 0.8633 |
| Hypothyroidism | Polymyositis | rs11406335 | 13 | 99934479 | G | T | G | T | -0.057 | 0.0103 | 3.44E-08 | 0.9867 | 0.1328 | 0.9867 |
| Hypothyroidism | Polymyositis | rs114285740 | 1 | 1.08E+08 | C | G | C | G | 0.1669 | 0.0301 | 3.06E-08 | 0.005996 | 0.3402 | 0.005996 |
| Hypothyroidism | Polymyositis | rs11675342 | 2 | 1407628 | T | C | T | C | 0.0906 | 0.01 | 1.4E-19 | 0.4096 | 0.1308 | 0.4096 |
| Hypothyroidism | Polymyositis | rs11875260 | 18 | 77179239 | G | A | G | A | 0.0751 | 0.0135 | 2.54E-08 | 0.6584 | 0.1723 | 0.6584 |
| Hypothyroidism | Polymyositis | rs12117927 | 1 | 2.37E+08 | A | C | A | C | 0.0627 | 0.0105 | 2.29E-09 | 0.04784 | 0.1351 | 0.04784 |
| Hypothyroidism | Polymyositis | rs12379417 | 9 | 1.27E+08 | A | G | A | G | 0.0583 | 0.0103 | 1.51E-08 | 0.476 | 0.1339 | 0.476 |
| Hypothyroidism | Polymyositis | rs12582330 | 12 | 1.04E+08 | T | G | T | G | -0.061 | 0.0109 | 2.05E-08 | 0.1254 | 0.1393 | 0.1254 |
| Hypothyroidism | Polymyositis | rs12593201 | 15 | 38844106 | A | G | A | G | 0.0905 | 0.0112 | 7.69E-16 | 0.1979 | 0.1471 | 0.1979 |
| Hypothyroidism | Polymyositis | rs12984428 | 19 | 50199616 | A | G | A | G | -0.0659 | 0.0102 | 1.11E-10 | 0.626599 | 0.1348 | 0.626599 |
| Hypothyroidism | Polymyositis | rs13090803 | 3 | 1.06E+08 | T | G | T | G | 0.0829 | 0.0128 | 9E-11 | 0.596 | 0.1872 | 0.596 |
| Hypothyroidism | Polymyositis | rs13109179 | 4 | 10727528 | A | G | A | G | 0.0647 | 0.01 | 9.42E-11 | 0.1588 | 0.1301 | 0.1588 |
| Hypothyroidism | Polymyositis | rs1364450 | 6 | 1.09E+08 | C | A | C | A | 0.0886 | 0.0139 | 1.97E-10 | 0.2041 | 0.1766 | 0.2041 |
| Hypothyroidism | Polymyositis | rs142997491 | 16 | 50729820 | G | A | G | A | 0.2385 | 0.0412 | 7.02E-09 | 0.773101 | 0.4393 | 0.773101 |
| Hypothyroidism | Polymyositis | rs1432806 | 5 | 1.09E+08 | G | A | G | A | 0.0583 | 0.0105 | 2.89E-08 | 0.6598 | 0.1401 | 0.6598 |
| Hypothyroidism | Polymyositis | rs1479565 | 5 | 76544157 | A | G | A | G | 0.0975 | 0.0101 | 7.53E-22 | 0.3994 | 0.1307 | 0.3994 |
| Hypothyroidism | Polymyositis | rs1534430 | 2 | 12644736 | T | C | T | C | -0.086 | 0.0101 | 1.44E-17 | 0.596499 | 0.1313 | 0.596499 |
| Hypothyroidism | Polymyositis | rs187707293 | 4 | 1.8E+08 | A | T | A | T | 0.2419 | 0.044 | 3.99E-08 | 0.7364 | 0.4778 | 0.7364 |
| Hypothyroidism | Polymyositis | rs2111485 | 2 | 1.63E+08 | G | A | G | A | 0.0813 | 0.0102 | 1.43E-15 | 0.1656 | 0.1311 | 0.1656 |
| Hypothyroidism | Polymyositis | rs2114702 | 14 | 81405135 | A | T | A | T | 0.07 | 0.0111 | 3E-10 | 0.1174 | 0.1576 | 0.1174 |
| Hypothyroidism | Polymyositis | rs2234167 | 1 | 2494330 | A | G | A | G | 0.0825 | 0.015 | 3.75E-08 | 0.1484 | 0.2001 | 0.1484 |
| Hypothyroidism | Polymyositis | rs2247314 | 6 | 1.67E+08 | C | T | C | T | -0.086 | 0.0104 | 1.06E-16 | 0.4267 | 0.1345 | 0.4267 |
| Hypothyroidism | Polymyositis | rs229528 | 22 | 37581677 | T | C | T | C | 0.0903 | 0.01 | 2.31E-19 | 0.4625 | 0.1318 | 0.4625 |
| Hypothyroidism | Polymyositis | rs2412976 | 22 | 30567907 | G | C | G | C | 0.0637 | 0.0103 | 5.47E-10 | 0.07453 | 0.139 | 0.07453 |
| Hypothyroidism | Polymyositis | rs2445608 | 8 | 1.28E+08 | A | G | A | G | -0.0593 | 0.0101 | 3.79E-09 | 0.052561 | 0.1326 | 0.052561 |
| Hypothyroidism | Polymyositis | rs244685 | 5 | 1.33E+08 | G | T | G | T | -0.0858 | 0.0132 | 7.06E-11 | 0.7001 | 0.1485 | 0.7001 |
| Hypothyroidism | Polymyositis | rs2921053 | 8 | 8319963 | C | G | C | G | -0.0599 | 0.0101 | 3.36E-09 | 0.9835 | 0.2315 | 0.9835 |
| Hypothyroidism | Polymyositis | rs2988277 | 1 | 1.67E+08 | T | C | T | C | 0.0593 | 0.0106 | 2.49E-08 | 0.777301 | 0.1476 | 0.777301 |
| Hypothyroidism | Polymyositis | rs307558 | 3 | 12095130 | A | G | A | G | -0.0688 | 0.0119 | 8.01E-09 | 0.468 | 0.1533 | 0.468 |
| Hypothyroidism | Polymyositis | rs3087243 | 2 | 2.05E+08 | A | G | A | G | -0.1466 | 0.0102 | 4.77E-47 | 0.4309 | 0.1372 | 0.4309 |
| Hypothyroidism | Polymyositis | rs3118469 | 10 | 6101129 | T | A | T | A | 0.0803 | 0.0106 | 3.82E-14 | 0.079811 | 0.1345 | 0.079811 |
| Hypothyroidism | Polymyositis | rs3184504 | 12 | 1.12E+08 | C | T | C | T | -0.1734 | 0.0102 | 7.5E-65 | 0.1105 | 0.1313 | 0.1105 |
| Hypothyroidism | Polymyositis | rs34536443 | 19 | 10463118 | C | G | C | G | -0.1863 | 0.0263 | 1.46E-12 | 0.8896 | 0.3737 | 0.8896 |
| Hypothyroidism | Polymyositis | rs3775291 | 4 | 1.87E+08 | T | C | T | C | -0.0649 | 0.0108 | 1.65E-09 | 0.8312 | 0.1389 | 0.8312 |
| Hypothyroidism | Polymyositis | rs434294 | 5 | 1.03E+08 | G | A | G | A | -0.0683 | 0.0109 | 3.36E-10 | 0.1726 | 0.1387 | 0.1726 |
| Hypothyroidism | Polymyositis | rs4409785 | 11 | 95311422 | C | T | C | T | 0.1069 | 0.0133 | 8.04E-16 | 0.451 | 0.1737 | 0.451 |
| Hypothyroidism | Polymyositis | rs4529854 | 10 | 64043975 | T | C | T | C | -0.0768 | 0.0107 | 6.56E-13 | 0.660201 | 0.1448 | 0.660201 |
| Hypothyroidism | Polymyositis | rs4835534 | 4 | 1.5E+08 | C | T | C | T | -0.1421 | 0.0132 | 7.06E-27 | 0.769899 | 0.1981 | 0.769899 |
| Hypothyroidism | Polymyositis | rs5912815 | X | 78466147 | G | T | G | T | -0.0511 | 0.0084 | 1.05E-09 | 0.5467 | 0.1156 | 0.5467 |
| Hypothyroidism | Polymyositis | rs61759532 | 17 | 7240391 | T | C | T | C | 0.0905 | 0.0122 | 1.42E-13 | 0.1692 | 0.1657 | 0.1692 |
| Hypothyroidism | Polymyositis | rs61877856 | 11 | 617378 | T | C | T | C | -0.0658 | 0.0115 | 1.14E-08 | 0.5635 | 0.1535 | 0.5635 |
| Hypothyroidism | Polymyositis | rs6679677 | 1 | 1.14E+08 | A | C | A | C | 0.3637 | 0.0159 | 2.4E-115 | 2.5E-05 | 0.1905 | 2.5E-05 |
| Hypothyroidism | Polymyositis | rs6908626 | 6 | 91005743 | T | G | T | G | 0.1441 | 0.0141 | 2.04E-24 | 0.1867 | 0.2037 | 0.1867 |
| Hypothyroidism | Polymyositis | rs7030280 | 9 | 1.01E+08 | T | C | T | C | 0.2075 | 0.0108 | 1.02E-82 | 0.8271 | 0.1357 | 0.8271 |
| Hypothyroidism | Polymyositis | rs71508903 | 10 | 63779871 | T | C | T | C | 0.0934 | 0.0125 | 9.34E-14 | 0.705399 | 0.1678 | 0.705399 |
| Hypothyroidism | Polymyositis | rs7223956 | 17 | 45373844 | C | T | C | T | -0.0902 | 0.0144 | 4.27E-10 | 0.3363 | 0.1853 | 0.3363 |
| Hypothyroidism | Polymyositis | rs73192661 | 3 | 1.88E+08 | T | C | T | C | -0.1061 | 0.01 | 4.05E-26 | 0.767701 | 0.1332 | 0.767701 |
| Hypothyroidism | Polymyositis | rs736374 | 11 | 35266944 | A | G | A | G | 0.0832 | 0.0103 | 6E-16 | 0.372 | 0.135 | 0.372 |
| Hypothyroidism | Polymyositis | rs7441808 | 4 | 26090375 | G | A | G | A | 0.0766 | 0.0111 | 5.17E-12 | 0.042531 | 0.143 | 0.042531 |
| Hypothyroidism | Polymyositis | rs7488011 | 12 | 9925336 | T | C | T | C | 0.1052 | 0.0111 | 2.52E-21 | 0.4844 | 0.1479 | 0.4844 |
| Hypothyroidism | Polymyositis | rs7574865 | 2 | 1.92E+08 | G | T | G | T | -0.1321 | 0.0117 | 1.67E-29 | 0.7536 | 0.1538 | 0.7536 |
| Hypothyroidism | Polymyositis | rs7742626 | 6 | 1.36E+08 | C | T | C | T | 0.0686 | 0.0116 | 3.41E-09 | 0.9673 | 0.1716 | 0.9673 |
| Hypothyroidism | Polymyositis | rs78765971 | 1 | 1.08E+08 | G | C | G | C | 0.2444 | 0.0162 | 1.68E-51 | 0.4579 | 0.2049 | 0.4579 |
| Hypothyroidism | Polymyositis | rs79490353 | 13 | 28623048 | C | T | C | T | 0.2006 | 0.0349 | 8.82E-09 | 0.07975 | 0.5498 | 0.07975 |
| Hypothyroidism | Polymyositis | rs7990020 | 13 | 43044946 | C | A | C | A | 0.0577 | 0.0101 | 9.97E-09 | 0.792899 | 0.1335 | 0.792899 |
| Hypothyroidism | Polymyositis | rs853305 | 8 | 1.34E+08 | C | T | C | T | -0.0802 | 0.0111 | 4.4E-13 | 0.7104 | 0.1587 | 0.7104 |
| Hypothyroidism | Polymyositis | rs881858 | 6 | 43806609 | A | G | A | G | 0.0665 | 0.0108 | 8.46E-10 | 0.102 | 0.1398 | 0.102 |
| Hypothyroidism | Polymyositis | rs911760 | 9 | 5438435 | A | C | A | C | 0.0879 | 0.0125 | 1.95E-12 | 0.624701 | 0.1595 | 0.624701 |
| Hypothyroidism | Polymyositis | rs926103 | 1 | 1.57E+08 | C | T | C | T | -0.0678 | 0.0104 | 7.65E-11 | 0.2151 | 0.1331 | 0.2151 |
| Hypothyroidism | Polymyositis | rs9264277 | 6 | 31224667 | C | T | C | T | -0.0862 | 0.0111 | 9.03E-15 | 0.1699 | 0.1451 | 0.1699 |
| Hypothyroidism | Polymyositis | rs9271365 | 6 | 32586794 | G | T | G | T | 0.2484 | 0.0105 | 4.9E-123 | 0.1771 | 0.14 | 0.1771 |
| Hypothyroidism | Polymyositis | rs9277559 | 6 | 33056731 | C | T | C | T | -0.133 | 0.012 | 1.86E-28 | 0.1242 | 0.1605 | 0.1242 |
| Hypothyroidism | Polymyositis | rs9497965 | 6 | 1.49E+08 | T | C | T | C | 0.0827 | 0.0102 | 3.71E-16 | 0.5634 | 0.1371 | 0.5634 |
| Hypothyroidism | Polymyositis | rs9511151 | 13 | 24786576 | A | G | A | G | -0.0976 | 0.0106 | 3.08E-20 | 0.559399 | 0.136 | 0.559399 |
| Hypothyroidism | Polymyositis | rs9902341 | 17 | 40278522 | T | C | T | C | 0.0801 | 0.0129 | 4.68E-10 | 0.5772 | 0.1619 | 0.5772 |
